# Effects of Normothermic Machine Perfusion Conditions on Mesenchymal Stromal Cells

**DOI:** 10.3389/fimmu.2019.00765

**Published:** 2019-04-10

**Authors:** Jesus M. Sierra Parraga, Kaithlyn Rozenberg, Marco Eijken, Henri G. Leuvenink, James Hunter, Ana Merino, Cyril Moers, Bjarne K. Møller, Rutger J. Ploeg, Carla C. Baan, Bente Jespersen, Martin J. Hoogduijn

**Affiliations:** ^1^Department of Internal Medicine, Erasmus Medical Center, University Medical Center Rotterdam, Rotterdam, Netherlands; ^2^Nuffield Department of Surgical Sciences and Oxford Biomedical Research Centre, University of Oxford, Oxford, United Kingdom; ^3^Department of Clinical Immunology, Aarhus University Hospital, Aarhus, Denmark; ^4^Department of Renal Medicine, Aarhus University Hospital, Aarhus, Denmark; ^5^Department of Surgery – Organ Donation and Transplantation, University Medical Center Groningen, Groningen, Netherlands

**Keywords:** mesenchymal stromal cells, normothermic machine perfusion, kidney repair, endothelial cells, suspension conditions, perfusion fluid, cryopreservation

## Abstract

*Ex-situ* normothermic machine perfusion (NMP) of transplant kidneys allows assessment of kidney quality and targeted intervention to initiate repair processes prior to transplantation. Mesenchymal stromal cells (MSC) have been shown to possess the capacity to stimulate kidney repair. Therefore, the combination of NMP and MSC therapy offers potential to repair transplant kidneys. It is however unknown how NMP conditions affect MSC. In this study the effect of NMP perfusion fluid on survival, metabolism and function of thawed cryopreserved human (h)MSC and porcine (p)MSC in suspension conditions was studied. Suspension conditions reduced the viability of pMSC by 40% in both perfusion fluid and culture medium. Viability of hMSC was reduced by suspension conditions by 15% in perfusion fluid, whilst no differences were found in survival in culture medium. Under adherent conditions, survival of the cells was not affected by perfusion fluid. The perfusion fluid did not affect survival of fresh MSC in suspension compared to the control culture medium. The freeze-thawing process impaired the survival of hMSC; 95% survival of fresh hMSC compared to 70% survival of thawed hMSC. Moreover, thawed MSC showed increased levels of reactive oxygen species, which indicates elevated levels of oxidative stress, and reduced mitochondrial activity, which implies reduced metabolism. The adherence of pMSC and hMSC to endothelial cells was reduced after the thawing process, effect which was particularly profound in in the perfusion fluid. To summarize, we observed that conditions required for machine perfusion are influencing the behavior of MSC. The freeze-thawing process reduces survival and metabolism and increases oxidative stress, and diminishes their ability to adhere to endothelial cells. In addition, we found that hMSC and pMSC behaved differently, which has to be taken into consideration when translating results from animal experiments to clinical studies.

## Introduction

As the outcome of kidney transplantation has improved, the demand for kidney transplantation has increased and the donor organ pool to date is too small to supply the current need for transplant organs. The shortage in available donor kidneys (1) has led to the use of expanded criteria donor organs, that includes kidneys from older donors or from donors with hypertension, suboptimal kidney function or death resulting from stroke (2). This has resulted in a higher decline rate at time of offering and may also lead to a poorer outcome of the transplantation (3).

Currently, several techniques are being employed to improve the quality of expanded criteria kidneys and discarded kidneys to make them suitable for transplantation, including machine perfusion. Hypothermic machine perfusion of donor kidneys implies connection of the organ to a pump that perfuses the organ with a solution that provides the required components needed to maintain viability while also removing waste products released as a result of the metabolism and perfusion injury of the organ.

A more physiological way to assess viability of donor organs is continuous perfusion at normothermic temperature at 37°C with proper oxygenation and in the presence of necessary nutrients. A few years ago, the clinical feasibility and safety of 1 h normothermic machine perfusion (NMP) was demonstrated (4). Other groups decided to evaluate the feasibility of longer term NMP at 37°C, allowing more time to observe the kidney as well as intervene where possible. Recently, NMP has been successfully tested in a series of discarded donor kidneys for up to 24 h (5, 6). During NMP, using a bespoke red blood cell (RBC) enriched oxygenated and nutrient containing perfusate, the metabolism of the kidney resumes and allows monitoring during perfusion prior to transplantation (6, 7). Application of NMP for assessment and targeted intervention (8) to improve kidney quality is appealing and the effect of the therapy can potentially be monitored before the organ is transplanted. Thus, NMP is postulated as a promising platform to reduce kidney damage and initiate regeneration prior to transplantation.

Mesenchymal stromal cells (MSC) are multipotent cells which are found in adult tissues where they support function and repair (9). The International Society for Cellular Therapy has established the minimum criteria that a cell must meet to be considered an MSC: *in vitro* attachment to plastic, expression of several cell surface markers including CD29, CD44, CD90, the absence of endothelial and hematopoietic markers and the capacity to differentiate into cell types of mesodermal origin (10). MSC provide growth factors to progenitor cells that boost their regenerative processes (11, 12). More than 800 MSC-related studies are registered at http://clinicaltrials.gov on December 2018 and some of them have shown promising results in the treatment of kidney injury from different etiologies (13).

MSC are usually intravenously (IV) administered, which inevitably leads to accumulation in the lungs and poor delivery to target organs (14, 15). Obviously, delivery of MSC to the target organ while perfused on an *ex vivo* stand-alone circuit may overcome this dilemma. The idea to combine NMP and MSC therapy has generated an interest in the area of kidney transplantation (16–18). It is however unknown whether MSCs are compatible with the conditions of NMP.

In addition, a significant difference exists between preparation of therapeutic MSC for pre-clinical vs. clinical trials. In pre-clinical experiments, MSC are cultured *in vitro* and administered directly from the culture flask to laboratory animals when the cells are ready for infusion. In the human setting, large numbers of MSC are needed to treat a patient and cells are often produced at locations distant from the place of administration. This requires storage of cells in a frozen state and infusion following a delicate thawing process (13). Existing literature points out that frozen-thawed human MSC (hMSC) have an altered gene expression profile compared to cells directly retrieved from culture flasks (19), while it also has been shown that MSC immunoregulatory properties may be impaired by the freeze-thawing process (20). If the properties of MSC are impaired after the thawing process, this would mean that the results of human studies may not have the expected outcome.

It has been described that MSC from different species will exert the same actions through different mechanisms, which could affect the efficacy of MSC in various animal models (21, 22). Human and non-human primate MSC (23, 24) show marked similarities with respect to their biological properties, but it is unknown whether their therapeutic effects are comparable. Porcine models are very suitable for organ transplant and preservation studies due to the similarity in size and physiology between human and pig. It is however unknown whether MSC from human and pig behave in the same manner under NMP conditions.

With the questions above in mind, it is important to simulate conditions of NMP and assess their effect on MSC. We have evaluated the effect of the bespoke perfusate required for NMP in combination with the condition of fresh vs. frozen-thawed MSC in suspension using cells from porcine (pMSC) and from human (hMSC) origin.

## Materials and Methods

### Isolation and Culture of Human and Porcine MSC and Endothelial Cells

hMSC were isolated from subcutaneous adipose tissue from healthy human kidney donors (*n* = 5) that became available during kidney donation procedures after obtaining written informed consent as approved by the Medical Ethical Committee of the Erasmus University Medical Center Rotterdam (MEC-2006-190). pMSC were isolated from subcutaneous adipose tissue (*n* = 5) collected from male pigs, which were subjected to surgery for teaching purposes, as a waste product. hMSC and pMSC were isolated as described previously (25) and phenotypically characterized by the expression of CD29, CD44, CD90 and the absence of CD31 and CD45. Human umbilical vein endothelial cells (HUVEC) were purchased from Lonza (Basel, Switzerland) and porcine aortic endothelial cells (PAOEC) were purchased from Cell Applications Inc. (San Diego, CA, USA).

Both hMSC and pMSC were cultured in minimum essential medium-α (MEM-α) (Sigma Aldrich, St. Louis, MO, USA) supplemented with penicillin (100 IU/ml), streptomycin (100 mg/ml) (1% P/S; Lonza), 2 mM L-glutamine (Lonza) and 15% fetal bovine serum (FBS; Lonza). HUVEC were cultured in endothelial growth medium 2 (PromoCell, Heidelberg, Germany). PAOEC were cultured in porcine endothelial cell media (Cell Applications, Inc.). MSC were used at passage 3-6, HUVEC were used at passage 4-8 and PAOEC at passage 3-6.

### Perfusion Fluid

The perfusion fluid was a RBC-based solution with albumin as colloid adapted from NMP experiments used by several groups and allowing stable NMP of kidneys (4, 26, 27). The composition of the perfusion fluid is listed in Table 1.

**Table 1:** Composition of perfusion fluid.

Red blood cells (Hematocrit 0.4 L/L)
Sodium (94.3 mmol/L)
Calcium (1.46 mmol/L)
Potassium (1.48 mmol/L)
Lactate (5.33 mmol/L)
Bicarbonate (26 mmol/L)
Albumin (19.1 g/L)
Glucose (2.93 mmol/L)
Mannitol (15.87 mg/L)
Creatinine (109.5 mg/L)
Amoxicillin (43.5 mmol/L)
Clavulanic Acid (16.1 mmol /L)

### Survival of MSC in Perfusion Fluid

MSC were trypsinized from the culture flasks at 90% confluency or thawed after cryopreservation and re-suspended either in complete culture medium or perfusion fluid at a concentration of 500,000 MSC/ml. MSC were incubated in perfusion fluid in polypropylene tubes to avoid attachment of MSC to plastic. After 30 min or 2 h in suspension, MSC were submitted to a RBC lysis process to remove the large amount of RBC present in perfusion fluid. MSC incubated in suspension with culture medium were also subjected to RBC lysis to treat both groups in the same way. Briefly, 3 ml of red blood cell lysis buffer (Invitrogen, Carlsbad, CA, USA) was added to MSC and incubated for 20 min at room temperature (RT). MSC were then washed with PBS and stained with Annexin-V (PE) and ViaProbe (PercP) to assess the number of early and late apoptotic cells. Perfusion fluid was also added to attached MSC and incubated for the same time and then trypsinized and stained as mentioned. Cells were analyzed by flow cytometry (FACS Canto II, BD Biosciences, NJ, USA) and data were analyzed using Kaluza Analysis 1.5a (Beckman Coulter, Brea, CA, USA).

### Mitochondrial Activity and Oxidative Stress of MSC

MSC metabolic activity was measured by a colorimetric assay based on the reduction of XTT [2,3-Bis-(2-Methoxy-4-Nitro-5-Sulfophenyl)-2H-Tetrazolium-5-Carboxanilide] by nicotinamide adenine dinucleotide (NADH) (ThermoFisher, Manhattan, NY, USA). The reagent is reduced by NADH produced during mitochondrial metabolism which results in a color change of the XTT reagent detectable by a spectrophotometer. The concentration of the reagent is measured by absorbance measured at a wavelength of 450–500 nm. This assay was performed on MSC that were in suspension for 30 and 120 min in perfusion fluid or culture medium and on attached MSC. In addition, oxidative stress of MSC was measured using CellRox reagent (ThermoFisher) according to the manufacturer's manual. CellRox is oxidized by reactive oxygen species (ROS) and emits a fluorescent signal that is measured by flow cytometry.

### Proliferation of MSC

In order to assess the effects of perfusion fluid on cell proliferation, MSC were fluorescently labeled with carboxyfluorescein succinimidyl ester (CFSE) (ThermoFisher) which was added to the cells at a concentration of 5 mM. The cells were then incubated at 37°C for 15 min in the dark. Staining was stopped by the addition of twice the volume of FBS-containing culture medium and incubated for 5 min at RT. Cells were washed and exposed to perfusion fluid for 30 and 120 min at 37°C. After incubation with perfusion fluid, MSC were seeded in a 6-well-plate with regular culture medium and proliferation was measured at 24, 48, and 72 h by flow cytometry.

### Release of Cytokines

A custom-made Luminex Multiplex Assay (R&D Systems, Minneapolis, MN, USA) was designed to measure the release of the following cytokines and growth factors by hMSC after incubation with perfusion fluid:

Angiopoietin-1 (ANG-1), Angiopoietin-2 (ANG-2), epidermal growth factor (EGF), hepatocyte growth factor (HGF), interferon-gamma (IFN-γ), interleukin 10 (IL-10), interleukin 6 (IL-6), monocyte chemoattractant protein 1 (MCP-1), programmed death ligand 1 (PD-L1), platelet derived growth factor AA (PDGFAA), Thrombospondin-2, tissue inhibitor of metalloproteases 1 (TIMP-1), tumor necrosis factor alpha (TNF-α), soluble tumor necrosis factor receptor 1 (sTNF-RI) and vascular endothelial growth factor (VEGF).

Subconfluent (90%) cultures of hMSC were washed and cells were incubated in perfusion fluid for 30 and 120 min. Wells were washed again and culture medium was added. After 24 h supernatants were retrieved and the array was performed according to the manufacturer's protocol. Fluorescence was measured on a Luminex 100/200 system (Luminex, Austin, TX, USA) using Xponent software. Due to the lack of pig-specific reagents this assay was only performed in with hMSC.

### Adhesion of MSC to Endothelial Cells

Confluent monolayers of endothelial cells were cultured in 24-well-plates. Culture medium was replaced by either a 1:1 mix of MSC and endothelial cell (EC) medium or perfusion fluid. 200,000 MSC were fluorescently-labeled with PKH26 (Sigma) and added to each well. After 10, 30, 60, 120, and 240 min supernatant was removed and wells were washed to eliminate all non-adherent MSC. Attached cells were trypsinized and analyzed by flow cytometry. Fluorescent signal detected by flow cytometry allowed the determination of the percentage of MSC attached.

## Results

### Cryopreserved MSC Show Reduced Survival in Perfusion Fluid and Medium

**Figure 1:**
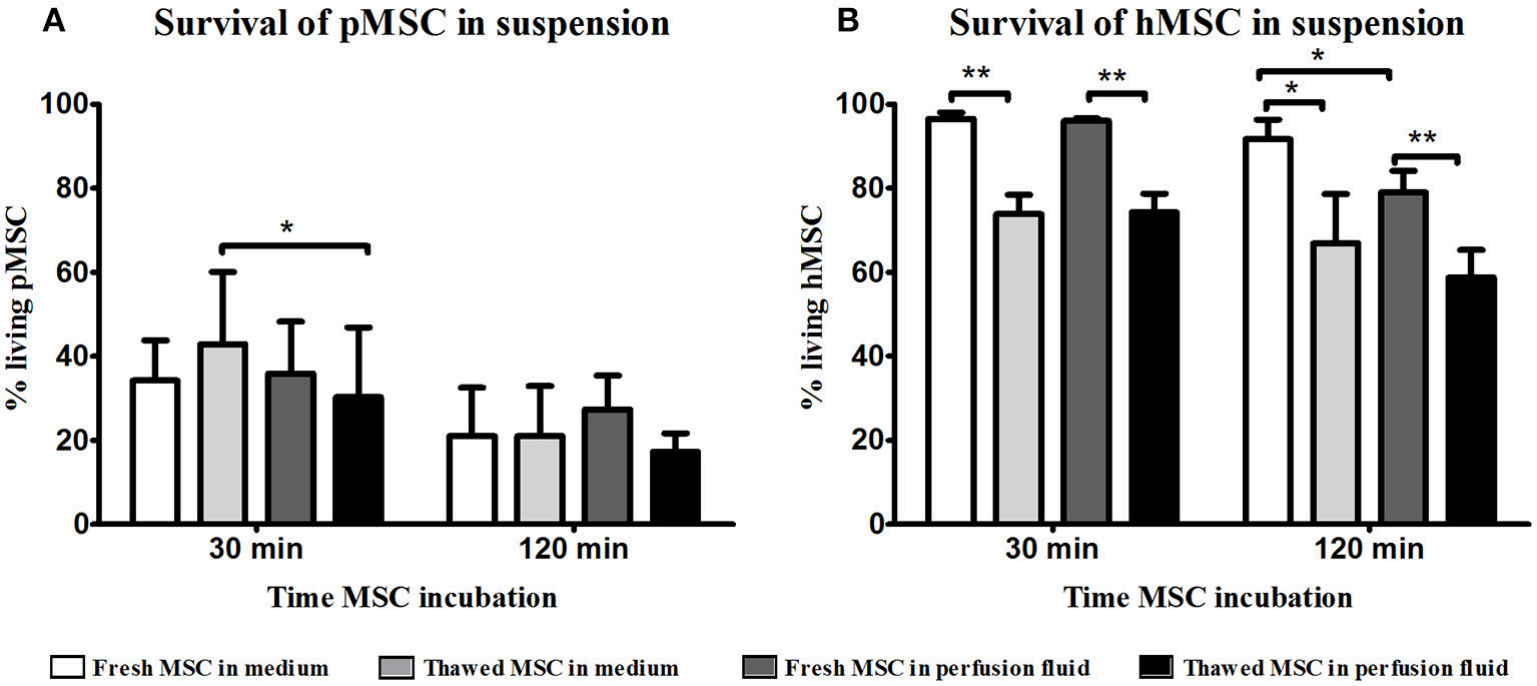
Effect of perfusion fluid on survival of fresh and thawed pMSC and hMSC in suspension. **(A)** Perfusion fluid had minimal effect on pMSC survival compared to medium. **(B)** Perfusion fluid had minimal effects on hMSC survival. Thawed hMSC showed a lower survival in suspension compared to fresh hMSC. Perfusion fluid reduced survival of hMSC after 2 h (*n* = 5). Results are shown as means ± SD. *p < 0.05; **p < 0.01.

To examine the survival of MSC, fresh and cryopreserved MSC were incubated in suspension in perfusion fluid. Survival rates of freshly cultured and thawed pMSC were <40% after 30 min and decreased to 30% after 2 h in culture medium. Perfusion fluid had no negative impact on pMSC survival compared to culture medium except on thawed MSC after 30 min in perfusion (Figure 1A). hMSC were more resistant to suspension conditions than pMSC. Freshly cultured hMSC showed more than 95% survival in medium and in perfusion fluid. However, a significant decrease in survival to approximately 70% was observed for cryopreserved hMSC in perfusion fluid and in culture medium compared to fresh MSC. Perfusion fluid reduced survival of fresh hMSC after 2 h only minimally when compared to regular culture medium (Figure 1B).

### MSC Show Impaired Adhesion to Endothelial Cells in Perfusion Fluid

**Figure 2:**
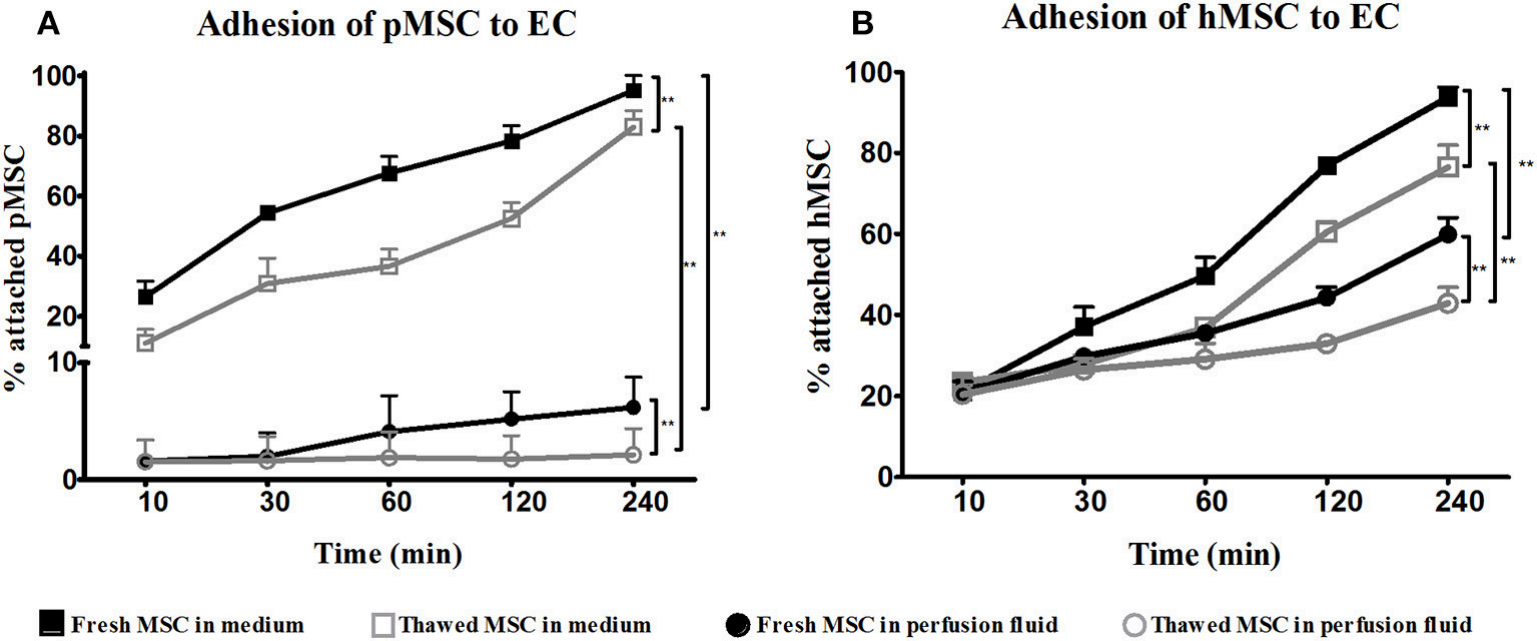
Adhesion of fresh and cryopreserved pMSC and hMSC to EC in medium and perfusion fluid. **(A)** pMSC attachment to PAOEC over time in culture medium. pMSC show reduced binding to PAOEC in perfusion fluid. Thawed pMSC showed a further reduced ability to bind to EC in perfusion fluid. **(B)** Thawed hMSC showed reduced attachment to HUVEC compared to fresh hMSC either in culture medium or perfusion fluid (*n* = 5). Results are shown as means ± SD. **p < 0.01.

To assess the function of surviving MSC in perfusion fluid, the capacity of MSC to adhere to EC was tested. In culture medium, fresh and thawed pMSC started to attach to PAOEC already 10 min after seeding. After 4 h, almost 100% of freshly cultured pMSC were attached to PAOEC while only 80% of thawed pMSC adhered (Figure 2A). Perfusion fluid strongly reduced the capacity of pMSC to attach to PAOEC regardless if they were fresh or thawed cells. Fresh hMSC adhesion to HUVEC was higher than 95% after 4 h in culture medium. In perfusion fluid, 60% of fresh hMSC were able to attach after 4 h. Cryopreserved hMSC showed 80% attachment in culture medium after 4 h and <50% of cryopreserved hMSC attached to HUVEC in perfusion fluid (Figure 2B). In general, thawed MSC showed a decreased capacity to attach to EC compared to fresh MSC, a difference that became more prominent in perfusion fluid.

### Thawed MSC Express Higher Levels of ROS

**Figure 3:**
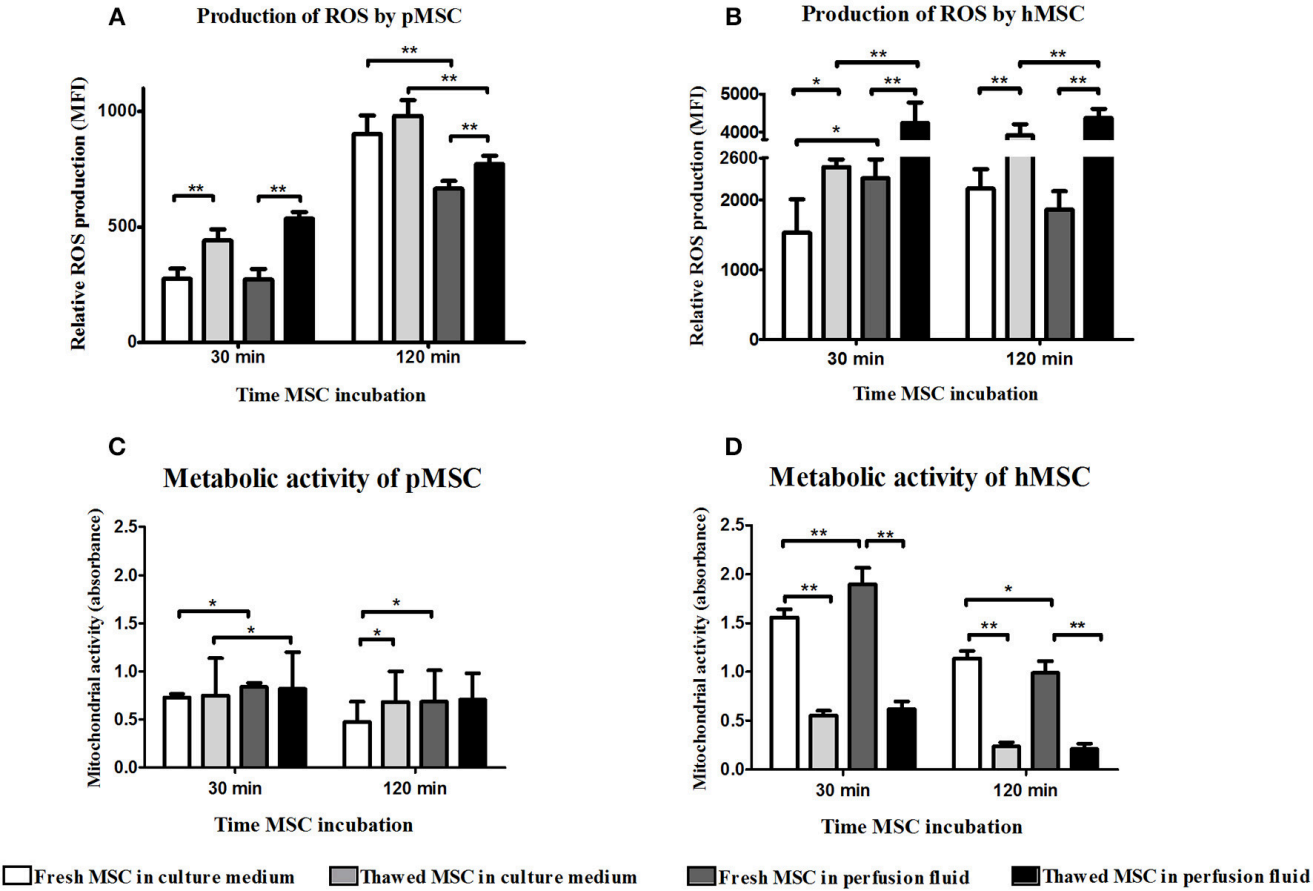
Perfusion fluid and thawing after cryopreservation increase ROS production and reduce metabolic activity in MSC. **(A)** ROS production in fresh and frozen-thawed pMSC suspended in culture medium or in perfusion fluid. **(B)** ROS production in fresh and in frozen-thawed hMSC suspended in culture medium or in perfusion fluid. **(C)** Metabolic activity measured by XTT reduction by NADH of fresh and frozen-thawed pMSC after 30 min and 2 h incubation in perfusion fluid. **(D)** Metabolic activity measured by XTT reduction by NADH of fresh and frozen-thawed hMSC after 30 min and 2 h incubation in perfusion fluid (*n* = 5). Results are shown as means ± SD. *p < 0.05; **p < 0.01.

The reduced adhesion of thawed MSC to EC could be explained by higher oxidative stress of MSC after the thawing process. The accumulation of ROS derived from the thawing process might induce damage in thawed MSC. An increased production of ROS by thawed pMSC and hMSC was observed at 30 min and 2 h after thawing both in medium and perfusion fluid (Figure 3A). Thawed hMSC produced a higher level of ROS than pMSC whereas ROS production in hMSC was boosted in perfusion fluid (Figure 3B). Thawed hMSC had elevated concentrations of ROS compared to fresh hMSC. These results indicate that freeze-thawing and perfusion fluid affects ROS production in pMSC and hMSC in the first hours after thawing.

### Metabolic Activity of Mitochondria Is Reduced in Thawed MSC

ROS production leads to mitochondrial damage which results in reduced metabolic activity of cells. It is possible that MSC survive in suspension but are less metabolically active, which may explain the different capacity of MSC to adhere to EC after thawing or in perfusion fluid. To determine the effect of perfusion fluid and the freeze-thawing procedure on MSC metabolic activity, the conversion of XTT to its reduced state by mitochondria was measured in MSC. pMSC mitochondrial activity showed to be very stable and was not affected by cryopreservation (Figure 3C). However, perfusion fluid induced a small increase in activity after 30 min. Fresh hMSC showed a 2-fold higher mitochondrial activity than pMSC. Furthermore, fresh hMSC were more active than their thawed counterparts. After 30 min in perfusion fluid, fresh hMSC showed increased mitochondrial activity compared to culture medium (Figure 3D).

### Freeze-Thawing Affects Proliferation of hMSC but Not pMSC

**Figure 4:**
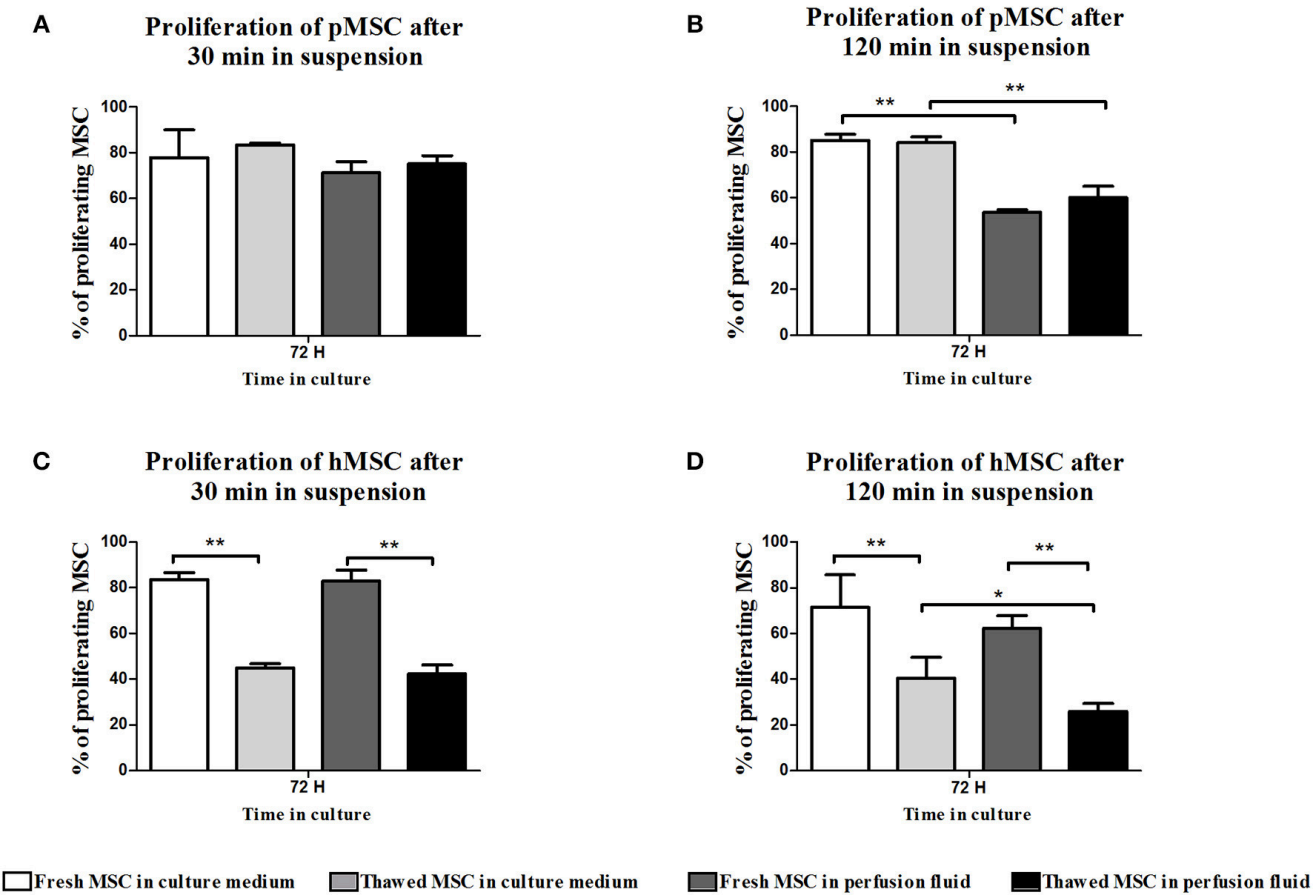
Effect of perfusion fluid and freeze-thawing on proliferation of MSC. pMSC and hMSC were incubated in perfusion fluid for 30 and 120 min and proliferation was measured after subsequent culturing in medium after 72 h. **(A)** pMSC proliferation was not affected by incubation in perfusion fluid for 30 min. **(B)** pMSC in perfusion fluid proliferated less than pMSC in culture medium. **(C,D)** Fresh hMSC proliferated more than thawed hMSC and perfusion fluid did not have an effect on fresh hMSC proliferation. Thawed cells in perfusion fluid were the least proliferative (*n* = 5). Results are shown as means ± SD. *p < 0.05; **p < 0.01.

Proliferation of MSC was measured after initial incubation in suspension in perfusion fluid or medium for 30 min and 120 min, followed by 72 h culture in culture medium. Incubation in perfusion fluid did not affect pMSC proliferation (Figures 4A,B). Freshly cultured hMSC proliferated more than thawed hMSC after incubation in perfusion fluid or medium (Figures 4C,D).

### Perfusion Fluid Increases the Proliferation of Attached MSC

**Figure 5:**
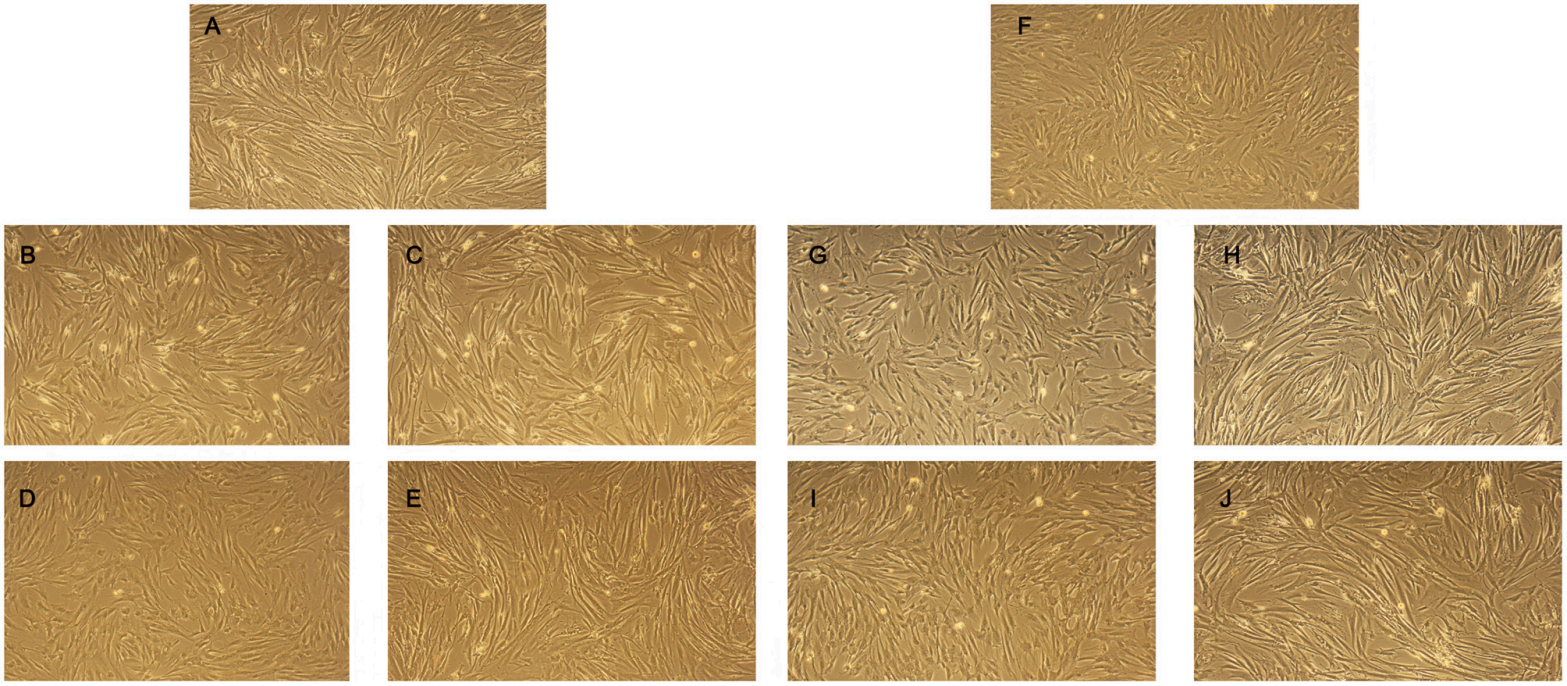
Effect of perfusion fluid on morphology of attached MSC. **(A)** pMSC in regular culture medium. **(B,C)** pMSC in culture medium or perfusion fluid, respectively for 30 min. **(D,E)** pMSC cultured in culture medium or perfusion fluid, respectively for 120 min. **(F)** hMSC in regular culture medium. **(G,H)** hMSC cultured in culture medium or perfusion fluid, respectively for 30 min. **(I,J)** hMSC cultured in culture medium or perfusion fluid, respectively for 120 min.

**Figure 6:**
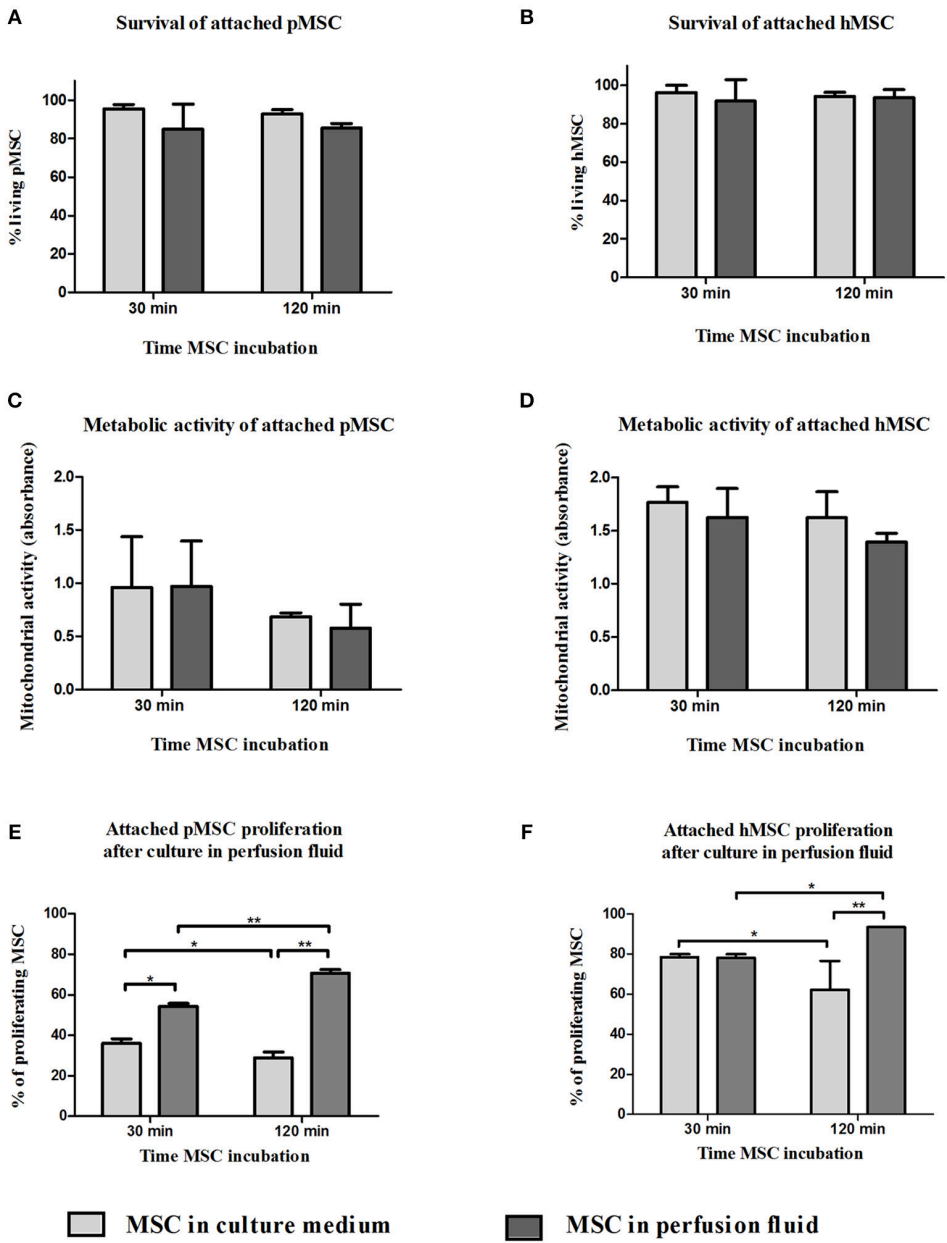
Effect of perfusion fluid on attached MSC. Survival of attached pMSC **(A)** and hMSC **(B)** after 30 min in perfusion fluid. **(C,D)** Metabolic activity of attached pMSC **(C)** and hMSC **(D)** after 30 min in perfusion fluid measured by reduction of XTT. **(E,F)** Proliferation of attached pMSC **(E)** and hMSC **(F)** after 30 in perfusion fluid. Cells were trypsinized and re-seeded in a culture flask. Proliferation after 24 h was determined by CFSE fluorescence (*n* = 5). Results are shown as means ± SD. *p < 0.05; **p < 0.01.

MSC are adherent tissue cells and the suspension conditions in the previous experiments may affect their phenotype and function. To examine how adherent MSC respond to perfusion fluid, morphology, survival, metabolic activity and proliferation of attached MSC were studied. The morphology of MSC in culture was not affected after 30 min or 2 h culture in perfusion fluid (Figures 5A–J). Adherent pMSC and hMSC showed increased survival in perfusion fluid compared to MSC in suspension. Survival of adherent pMSC was higher than 80% in culture medium and perfusion fluid (Figures 6A,B) compared to a maximum of 40% survival in suspension (Figure 1A). hMSC showed 78% survival after 2 h in suspension in perfusion fluid, but when they were attached, survival after 2 h was 93%. No differences in survival were observed between perfusion fluid and culture medium on attached MSC (Figures 6A,B). No significant effect of perfusion fluid on mitochondrial activity was observed for attached MSC, although there was a trend toward a decline in activity of pMSC and hMSC over time when cultured in perfusion fluid (Figures 6C,D). Pre-incubation of attached pMSC in perfusion fluid increased their proliferation after 24 h compared to culture medium. This effect was observed only after 120 min pre-incubation in perfusion fluid for hMSC (Figures 6E,F).

### Secretory Profile of MSC Is Not Affected by Culture in Perfusion Fluid

**Figure 7:**
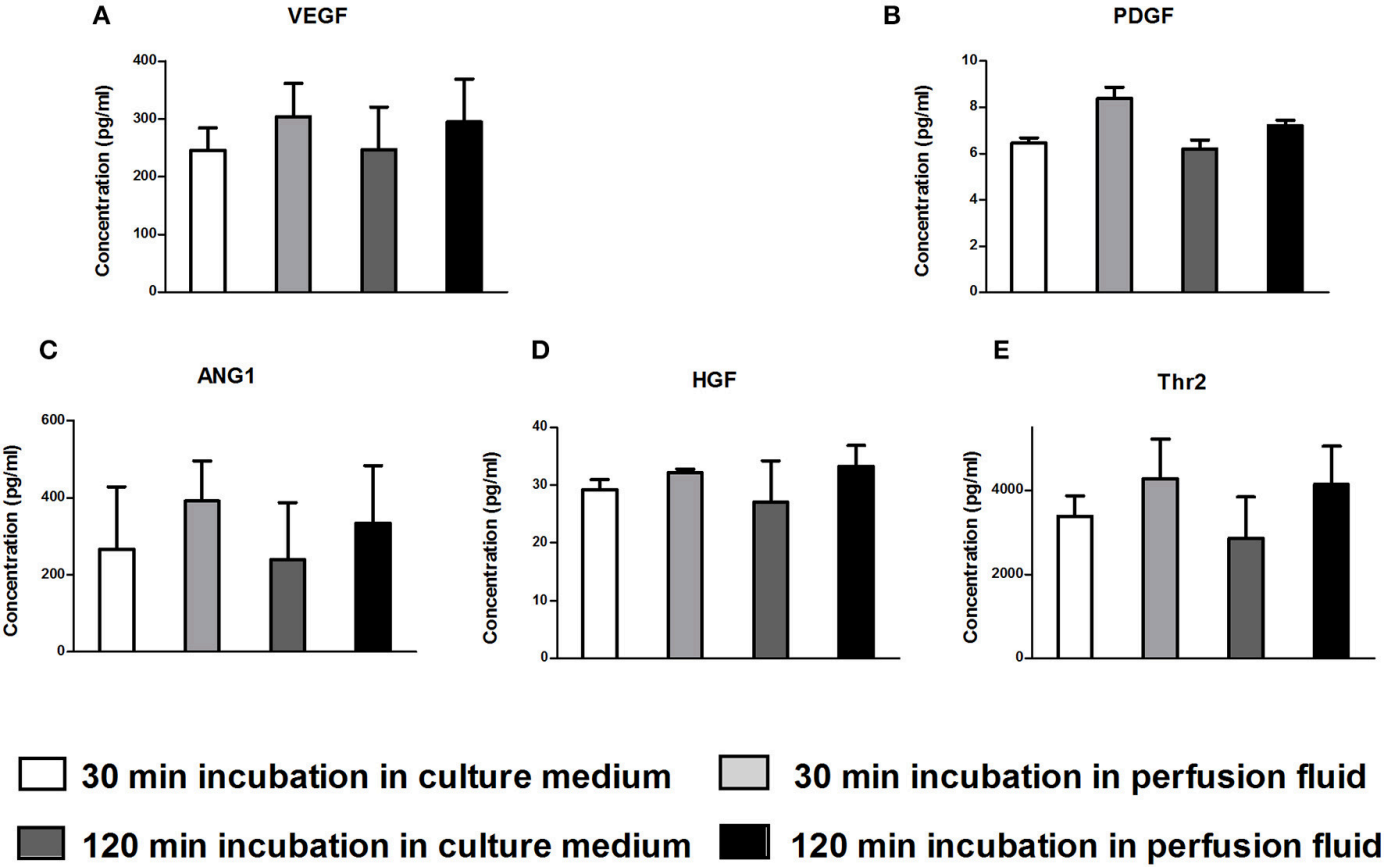
Production of angiogenic factors by hMSC in culture medium and perfusion fluid. MSC were incubated in perfusion fluid for 30 or 120 min, washed and replaced by culture medium for 24 h. The secretion of angiogenic and growth factors was not affected by perfusion fluid. **(A–E)** Concentration of secreted VEGF, PDGF, ANG1, HGF and Thr2, respectively (*n* = 5). Results are shown as mean ± SD.

**Figure 8:**
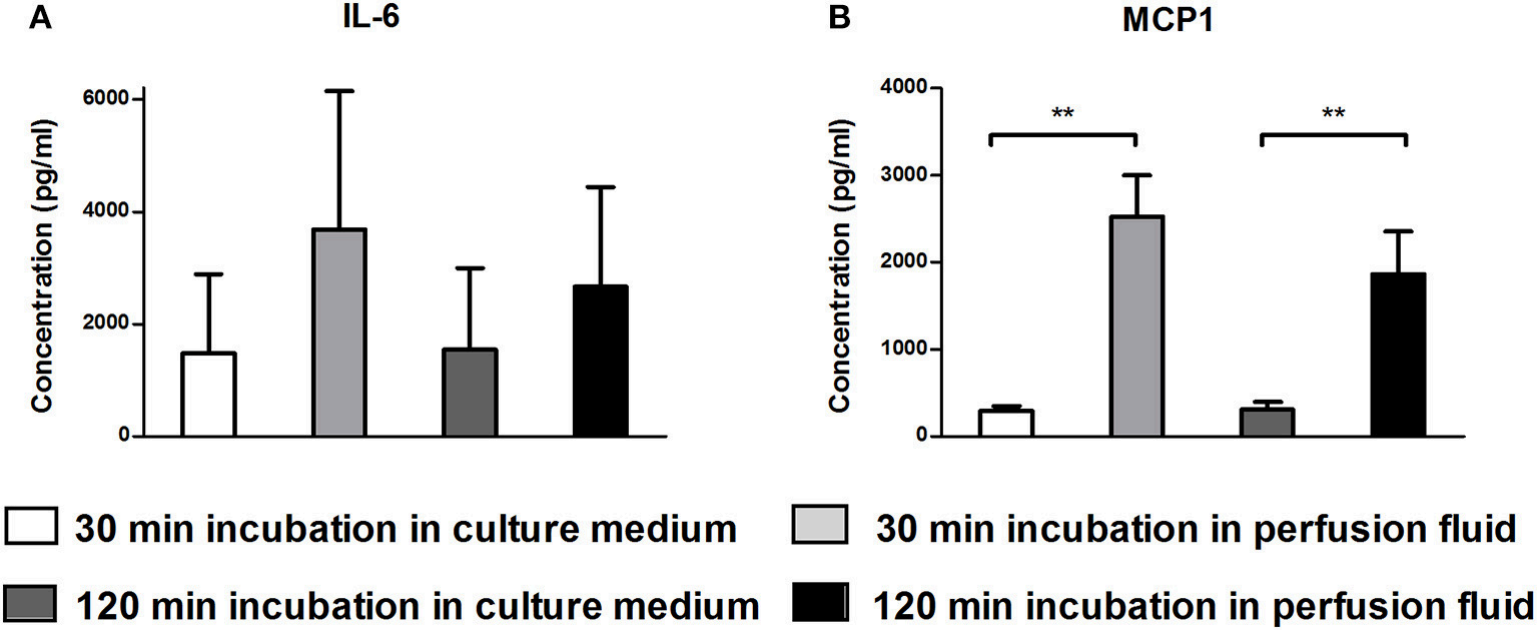
Production of inflammatory cytokines by hMSC in culture medium and perfusion fluid. MSC were incubated in perfusion fluid for 30 or 120 min, washed and replaced by culture medium for 24 h. **(A)** The secretion of IL-6 shows tendency to increase by perfusion fluid. **(B)** The secretion of MCP-1 by MSC is increased after incubation in perfusion fluid (*n* = 5). Results are shown as means ± SD. **p < 0.01.

The secretion of growth factors and cytokines is an important mechanism of action of MSC. To examine whether perfusion fluid would preserve the secretory profile of adherent hMSC and furthermore whether perfusion fluid induced an inflammatory response in hMSC, hMSC were incubated in perfusion fluid for 30 min or 2 h and growth factor and cytokine secretion was analyzed. We observed that the secretion of the angiogenic factors VEGF, PDGF, ANG-1, HGF and Thr2 was unaffected in perfusion fluid (Figures 7A–E). Inflammatory cytokines IL-6 and MCP-1 were increased 2-fold and 10-fold, respectively, in perfusion fluid (Figures 8A,B).

## Discussion

In the present work we have assessed the effect of a period of incubation in a perfusion fluid required for robust longer term *ex vivo* NMP of kidneys on MSC of both pig and human origin. Our work involved the use of cryopreserved MSC for logistic purposes, suspension conditions to deliver MSC via the renal artery using NMP and the use of an RBC-based perfusion fluid which may affect the survival and function of MSC. In order to mimic the conditions of a potential novel MSC therapy to stimulate the repair of injured kidneys while these are connected to NMP, MSC were thawed after cryopreservation and incubated in perfusion fluid in suspension. Actual infusion of MSC using NMP was not carried out as the purpose of the study was to assess the individual effect of each of the aforementioned conditions separately. In case that NMP conditions did not support MSC survival and function, future planned experimentation in the NMP setup could have been stopped, reducing economic and time costs. Nevertheless, the results of this study allow to take the next step and study MSC infusion through an NMP system, which is already planned to be carried out.

In the field of clinical MSC therapy, temporary cryopreservation and thawing along with vehicle solutions to deliver the therapy are important factors that can determine the success of MSC therapy (20, 28–30). The handling time until administration will have an impact on the survival and function of MSC. Survival of MSC is affected by the composition of storage media (31) and cytokine secretion profile of MSC can be altered, affecting MSC properties such as angiogenic potential (32). Our results show that the particular perfusion fluid used for our experiment is not detrimental for the secretion of angiogenic factors by hMSC.

The bespoke NMP perfusion fluid used in this study supports the survival of pig and human MSC. However, the function and metabolism of these cells are affected by the suspension conditions MSC were kept in. Specifically, perfusion fluid inhibited the adhesion of pMSC in suspension to PAOEC and hMSC to HUVEC. MSC have the capacity to adhere to endothelial cells *in vitro* under flow conditions as previously shown, especially when endothelial cells have been treated to recreate an inflammatory environment. Attachment of MSC to endothelial cells was shown to be reduced under flow compared to static conditions (33). Being in suspension in different clinically used storage solutions such as physiologic saline can influence the survival of MSC (31) and the composition of the solution that MSC are kept in modifies their metabolism and function (34, 35). Therefore, it can be deducted that the effect of MSC therapy delivered to renal grafts during *ex vivo* NMP will depend, among other factors, upon the composition of the perfusion fluid. A recent study perfused kidneys for 24 h using NMP. They infused MSC at different concentrations however, after 24 h they found 95% of infused MSC back in their perfusion solution (36), which indicate a very diminished adhesion capacity of MSC toward endothelial cells. Our results are consistent with existing literature and indicate that the composition of the perfusion fluid as well as the infusion process affects the functional properties and delivery efficiency of the final MSC product. Therefore, further knowledge need to be obtained regarding the effects of perfusion conditions on MSC delivery to the injured kidney.

Human and porcine MSC showed a negative response at several levels to cryopreservation, thawing and re-suspension in perfusion fluid. The effect of cryopreservation and thawing of hMSC has been a concern for the community as it can decrease the presumed efficacy of MSC therapy (19, 28, 30, 37). Our results confirmed this concern. It has recently been published that freezing-thawing MSC increases the production of ROS and compromises membrane stability and homeostasis in pMSC (38), which is also supported by our data. Viability of MSC is a key factor for treatments that require an active role of MSC. However, inactive MSC have been shown to retain their immunomodulatory properties (14, 39, 40). Therefore, the aim of MSC therapy dictates the required characteristics of MSC.

*In vivo*, MSC are tissue resident cells and are found in perivascular niches in a wide variety of tissues (41–43). *In vitro*, MSC are strictly grown as adherent cells, and for this reason MSC are cultured allowing plastic adherence to expand them (10). We demonstrated that the poor performance of MSC in perfusion fluid was primarily due to the fact that they were in suspension. Survival of both pig and human MSC cultured in adherent conditions in perfusion fluid was higher than that of MSC kept in suspension, suggesting a protective effect of adherence. Mitochondrial activity, however, was similar in MSC in suspension and attached, suggesting that MSC are active also in suspension. Proliferation of pMSC was increased after exposure to perfusion fluid which could be a response to a specific component of the perfusion fluid (31). These results suggest that the nature of MSC make them more vulnerable when they are in suspension and therefore, it should be minimized when they are administered as therapy. Being in suspension in perfusion is, however, a transient condition in the process of MSC delivery using NMP. Presumably, when MSC are delivered to the injured kidney, the damaged tissue microenvironment will help the MSC to be retained and produce regenerative factors as previously shown (44–46). We have shown that after being in contact with perfusion solution MSC can recover, proliferate and be metabolically active. In addition, the secretory profile of angiogenic factor by hMSC is not affected by perfusion fluid. Therefore, this is a promising result that hints MSC maintain their reparative potential after delivery using NMP.

Cryopreservation, thawing and suspension of MSC are inevitable conditions to infuse GMP-grade MSC to kidney transplants via NMP. These conditions are necessary to bridge differences in time and location between MSC preparation and NMP. The disadvantageous effect of these conditions on MSC has to be taken into account for the evaluation of the suitability of MSC therapy for NMP. In order to improve the viability of MSC in NMP conditions, the composition of the perfusion fluid may be adapted to provide better support for MSC survival. Another possibility is to recover MSC after thawing under favorable conditions to improve the resistance of MSC to NMP. An alternative would be to take the loss of MSC under NMP conditions into account and use higher numbers of cells, although the effect of administering large numbers of non-viable MSC to the kidney is uncertain.

Pre-clinical work in the field of transplantation is often performed using porcine models to better understand the possible behaviors of new therapies in patients. Therefore, we investigated MSC of porcine and human origin in order to determine if results can be translated. The response to the thawing process as well as to suspension conditions and perfusion fluid was quite different between MSC from both origins for the parameters studied. We are aware that differences could be related not only to species but also to age and gender, as well as site of adipose tissue harvesting from the donor. However, our results indicate that caution should be taken when interpreting *in vitro* studies with porcine cells toward the behavior of human cells. A safe translation from swine pre-clinical models to clinical studies is challenging when results are not reproducible between species.

Summarizing, NMP conditions will affect MSC but show sufficient support of their function and survival to consider MSC administration through NMP as a viable option in pursuit of a potentially beneficial cell therapy for the regeneration of injured organs. After these essential preliminary experiments, further study is now underway to determine the best way of joining these two exciting techniques in an optimal manner.

## Author Contributions

BJ, CB, ME, RP, and MH: conception of the study; JS, AM, and MH: designed the experiments; JS: performed the experiments and the analysis of the data; JS, AM, and MH: interpreted the data. All authors contributed to writing and reviewing the manuscript.

### Conflict of Interest Statement

The authors declare that the research was conducted in the absence of any commercial or financial relationships that could be construed as a potential conflict of interest.

## Abbreviations

EC: Endothelial cells
hMSC: Human mesenchymal stromal cells
HUVEC: Human umbilical cord endothelial cells
MSC: Mesenchymal stromal cells
NADH: Nicotinamide adenine dinucleotide
NMP: Normothermic machine perfusion
PAOEC: Porcine aortic endothelial cells
pMSC: Porcine mesenchymal stromal cells
ROS: Reactive oxygen species
XTT, 2: 3-Bis-(2-Methoxy-4-Nitro-5-Sulfophenyl)-2H-Tetrazolium-5-Carboxanilide.
